# Supplementary material for: DMRforPairs: identifying Differentially Methylated Regions between unique samples using array based methylation profiles
Source: BMC Bioinformatics. 2014 May 15;15:141. doi: 10.1186/1471-2105-15-141 (PMC4046028; doi:10.1186/1471-2105-15-141)
Supplement: Additional file 2 — DMRforPairs output for the comparison of A431-MCF7 and NA17018-NA17105. Please start from the HTML files in each folder. Available via the BMC Bioinformatics website. [file 1471-2105-15-141-S2.zip › 1394847754114233_MOESM2_ESM/A431_MCF7/figures/17681.pdf]

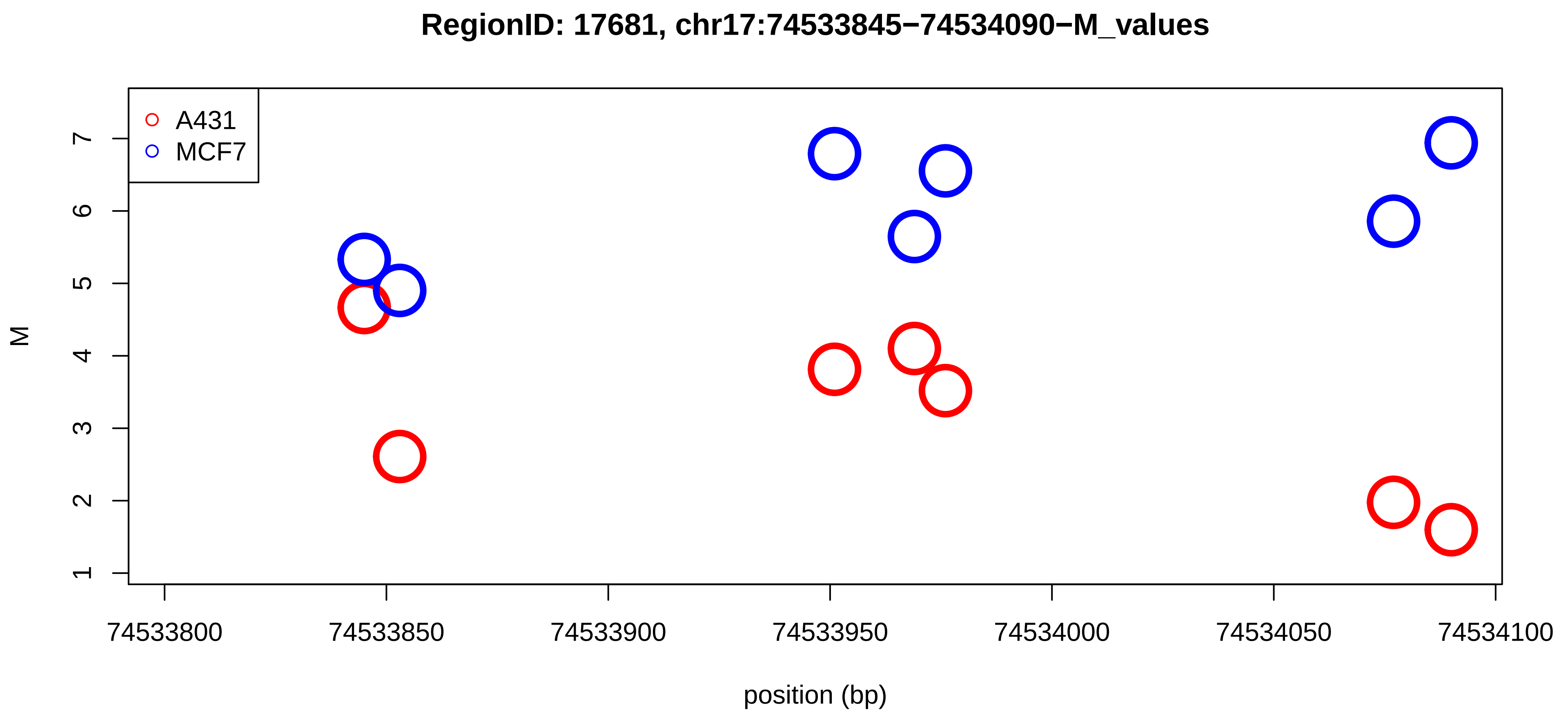
RegionID: 17681, chr17:74533845−74534090−M_values
A431
MCF7
M
1
2
3
4
5
6
7
74533800
74533850
74533900
74533950
74534000
74534050
74534100
position (bp)

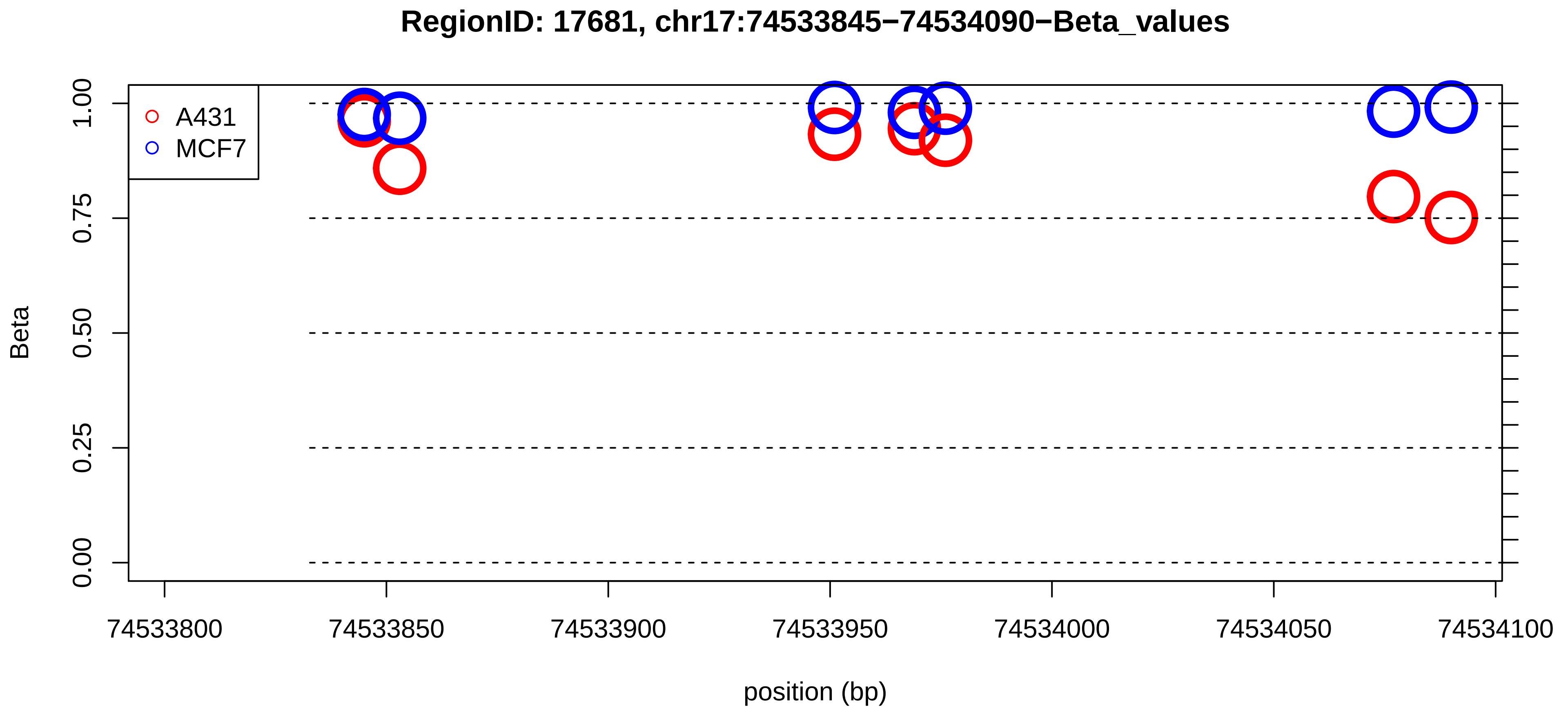
RegionID: 17681, chr17:74533845−74534090−Beta_values
A431
MCF7
Beta
0.00
0.25
0.50
0.75
1.00
74533800
74533850
74533900
74533950
74534000
74534050
74534100
position (bp)

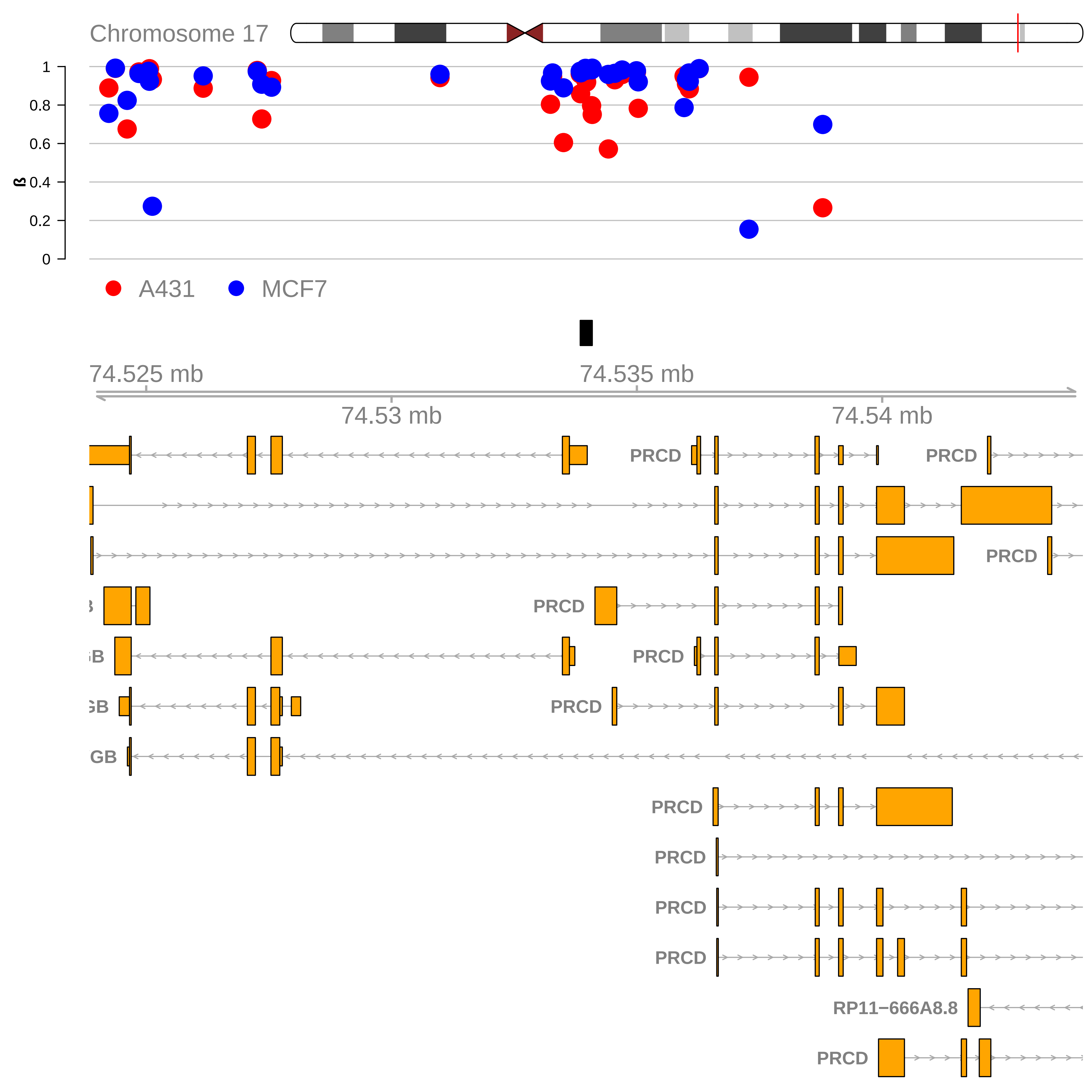

Chromosome 17
β
1
0.8
0.6
0.4
0.2
0
A431
MCF7
74.525 mb
74.535 mb
74.53 mb
74.54 mb
PRCD
PRCD
PRCD
PRCD
PRCD
PRCD
PRCD
PRCD
PRCD
PRCD
RP11−666A8.8
PRCD
